# Health system barriers to strengthening vaccine-preventable disease surveillance and response in the context of decentralization: evidence from Georgia

**DOI:** 10.1186/1471-2458-6-175

**Published:** 2006-07-05

**Authors:** David R Hotchkiss, Thomas P Eisele, Mamuka Djibuti, Eva A Silvestre, Natia Rukhadze

**Affiliations:** 1 Department of International Health and Development, Tulane University School of Public Health and Tropical Medicine, New Orleans, LA, USA; 2 Curatio International Foundation, Tbilisi, Georgia

## Abstract

**Background:**

A critical challenge in the health sector in developing countries is to ensure the quality and effectiveness of surveillance and public health response in an environment of decentralization. In Georgia, a country where there has been extensive decentralization of public health responsibilities over the last decade, an intervention was recently piloted to strengthen district-level local vaccine-preventable disease surveillance and response activities through improved capacity to analyze and use routinely collected data. The purpose of the study is 1) to assess the effectiveness of the intervention on motivation and perceived capacity to analyze and use information at the district-level, and 2) to assess the role that individual- and system-level factors play in influencing the effectiveness of the intervention.

**Methods:**

A pre-post quasi-experimental research design is used for the quantitative evaluation. Data come from a baseline and two follow-up surveys of district-level health staff in 12 intervention and 3 control Center of Public Health (CPH) offices. These data were supplemented by record reviews in CPH offices as well as focus group discussions among CPH and health facility staff.

**Results:**

The results of the study suggest that a number of expected improvements in perceived data availability and analysis occurred following the implementation of the intervention package, and that these improvements in analysis could be attributable to the intervention package. However, the study results also suggest that there exist several health systems barriers that constrained the effectiveness of the intervention in influencing the availability of data, analysis and response.

**Conclusion:**

To strengthen surveillance and response systems in Georgia, as well as in other countries, donor, governments, and other stakeholders should consider how health systems factors influence investments to improve the availability of data, analysis, and response. Linking the intervention to broader health sector reforms in management processes and organizational culture will be critical to ensure that efforts designed to promote evidence-based decision-making are successful, especially as they are scaled up to the national level.

## Background

There is broad consensus among health professionals that health information systems (HIS) should play an integral part of any health system [1-3]. Health information is essential in determining whether a health system is effective in detecting health problems, defining priorities, identifying solutions, and allocating resources to improve health outcomes [4,5]. With the formation of the Health Metrics Network, the Routine Health Information Network (RHINO) and the recently-established Ellison Institute, the issue of strengthening HIS in low- and middle-income countries has received an unprecedented amount of attention in recent years.

One essential source of health-related information is surveillance and response systems focused on epidemic and vaccine-preventable disease (VPD). VPD surveillance systems have the potential to improve the efficiency and effectiveness of VPD prevention and control programs by targeting interventions and documenting their effect on the population. The performance of surveillance and response systems depends not only on the availability and quality of health information, but also on the demand for information by health policy makers and managers. Two key dimensions of demand are the desire to analyze information and the desire to use of information for responses or actions. In a decentralized context, the process of analysis and response should be driven by the local-level surveillance and response program manager's desire to use data to improve decision-making. In order for this to occur, surveillance information must be perceived as useful for decision-making, and expectations for analysis, interpretation, and translation into action must be clearly laid out.

Effectively conducting VPD surveillance and response is particularly challenging in countries that have introduced health sector decentralization reforms [6,7]. There are a number of reasons why decentralization might have substantial implications for HIS. First, surveillance systems may not perform well in a decentralized context if local epidemiological health staff lack the capacity to carry out devolved surveillance and response functions (i.e. the collection, analysis, and actual use of information for public health responses) previously carried out by national level staff and if there is low demand for health information at the local government level. Second, demand for information may be limited if surveillance systems designed for centralized systems are unaligned with the needs of local users. This mismatch between the availability of information and user needs can be further exacerbated if subsequent efforts to reform the surveillance system are "data led" rather than "action led" [8]. Third, factors that operate at the health system level, such as limited local resources, low health worker motivation due to a lack of incentives, inadequate transportation and communication, and weak accountability, also play greater roles in limiting system performance. Many of these concerns have led some experts to conclude that "a critical challenge that is faced in the health sector in developing countries is to ensure the quality and effectiveness of public health surveillance and response in an environment of decentralization" [7].

Most previous research that evaluates public health surveillance systems focus on the assessment of one or two major attributes of surveillance systems such as sensitivity and timeliness [9]. However, there is relatively little research that investigates the effectiveness of strategies to strengthen the capacity and motivation of health workers to carry out public health surveillance and response within a decentralized country context. In fact, in an article on public health surveillance that appeared in the World Bank's recently published Disease Control Priorities in Developing Countries (Second Edition), Nsubuga et al state that the most important research question for surveillance that needs to be addressed is how to go about developing and maintaining "a cadre of competent, motivated surveillance and response workers in developing countries" [7]. Given the increased concern regarding the consequences of decentralization on public health surveillance and response activities, attempts to answer this question in countries where surveillance and response functions have been devolved are of particular relevance.

The purpose of this paper is help fill this research gap by 1) assessing how health worker motivation, perceived capacity, and actual use of information for decision making of district-level epidemiological staff in Georgia have been influenced by a pilot VPD surveillance and response intervention, and 2) assessing the remaining barriers to improving the use of data for programmatic and epidemiological responses. Georgia is a middle-income country where there has been extensive decentralization of public health responsibilities over the last decade. Aimed to strengthen local VPD surveillance and response activities through improved analysis and use of routinely collected data, the intervention focused on clarifying roles and responsibilities at the rayon (district) level for analysis and outlining links to actions, by improving capacity, and by ensuring the availability of resources necessary for the non-personnel costs of disease outbreak investigation and selected monitoring functions. All of these efforts were intended to improve the value that health workers place on VPD surveillance information, resulting in improved capacity and motivation to analyze and use information for epidemiological responses, program management, and resource allocation decisions.

## Methods

### Intervention

**Figure 1:**
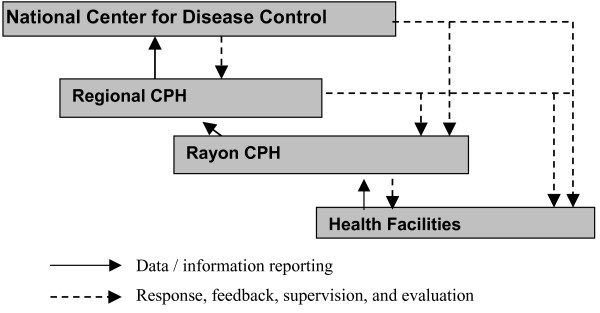
**Routine reporting channels of epidemiological surveillance system in Georgia**. Note: CPH = Center for Public Health

In 1995, Georgia embarked on an ambitious decentralization initiative that has had substantial effects on infectious disease surveillance and response activities. The Soviet-style sanitary-epidemiological system was divided into two separate entities – sanitary control and epidemiological service – and many surveillance and control functions, including many that pertain to surveillance data collection, analysis, and response, were delegated to the city and rayon government levels. As depicted in Figure 1, rayon-level Center for Public Health (CPH) offices collect information from health facilities and then report routine information to both regional CPH offices and the National Center of Disease Control on a monthly and annual basis.

The intervention evaluated in this study was implemented in the region of Imereti with the aim of improving the analysis of routinely-collected VPD surveillance data and resultant responses. In terms of population size, Imereti is the largest region in Georgia, making up 16% of the total population of 4.37 million in 2002 [10].

**Figure 2:**
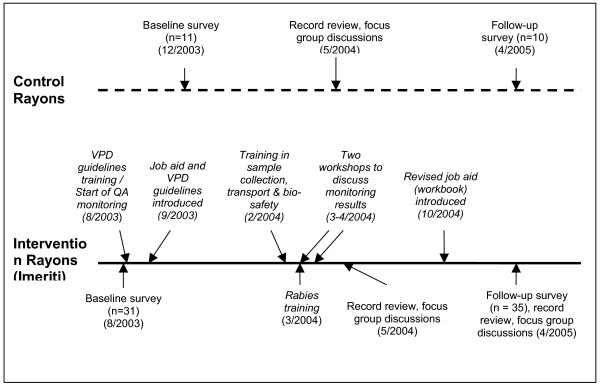
**Timeline of implementation and data collection activities in Imereti region and control areas**. Figure legend text.

The intervention was supported by United States Agency for International Development (USAID) through the Partners for Health Reform*plus *Project, and consists of both technical and behavioral components designed to standardize and facilitate analysis of VPD data and its translation into public health actions. The intervention components included: 1) surveillance guidelines for rayon level public health managers along with a surveillance handbook for health facility staff; 2) a job aid for rayon CPH offices to help guide analysis and use of VPD data, consisting of a workbook outlining required monthly analyses and appropriate response options; 3) training in the guidelines for both rayon CPH and facility staff; 4) on-the-job technical assistance; and 5) a financial reimbursement system for specified analysis and response activities. The intervention was uniformly implemented throughout all 12 rayons of the Imereti region of Georgia starting in August 2003 (Figure 2). Based on feedback and preliminary qualitative results, the job aid workbook was revised midway through the study in October 2004.

### Study design

The effectiveness of the intervention on perceived capacity and motivation was evaluated through the use of a longitudinal pre-post quasi-experimental research design, supplemented where possible with data from surveillance system record reviews and focus group discussions. A cohort of individuals responsible for VPD data analysis and response within CPH offices within all 12 rayons of the Imereti Region served as the intervention group. Individuals within CPH offices within three rayons outside the intervention area were selected to serve as controls to help validate any resultant changes in analysis and response within the intervention group. Because randomization was not feasible, the three control CPH offices (Senaki, Chokhatauri, and Tbilisi) were chosen for their similarity to the intervention rayons in surveillance motivation and performance (based on expert experience), location (mountainous vs. flat) and population composition (by age and sex).

Outcomes pertaining to analysis and response within intervention and control groups were measured among the same individuals at baseline and again one and half years later at follow-up after the implementation of the intervention package (Figure 2).

The Institutional Review Board of Tulane University's Health Sciences Center approved the protocol of the study. All participants gave informed written consent.

### Outcome indicators

Outcome measures were grouped into five topic areas that were hypothesized to improve as a result of the intervention: 1) availability of quality data from subordinate health facilities; 2) capacity to perform routine data analysis; 3) motivation to perform data analysis; 4) value of using analyzed data for decision-making; and 5) motivation to use analyzed data for decision-making. Likert scale questions at five levels, ranging from strongly disagree to strongly agree, were used to ascertain information for constructing outcome indicators. All outcomes are measured and analyzed at the individual level. The decision to focus on perceptions and motivation of rayon and health facility staff was based on the premise that improvements in self-efficacy and the perceived value information are necessary to improve how surveillance data are used to improve the performance of the vaccine preventable disease surveillance and response program.

While perceived motivation to analyze and use VPD data were ascertained from single questions, multiple Likert scale questions addressing the underlying constructs of the availability of data, capacity to analyze data and perceived value of using data for decision-making were grouped into composite indexes using a Cronbach coefficient alpha correlation analysis, based on baseline results (Table 1). Composite indices were obtained from the mean of combined Likert scale questions that had a Cronbach coefficient alpha raw score greater than 0.70.

### Sample size and data collection

Outcome measures were ascertained using a self-administered survey questionnaire to a cohort of public health professionals responsible for analysis and use of VPD data at CPH offices within intervention and control rayons at baseline and again at follow-up. The questionnaire consisted of Likert scale, yes/no and open-ended questions. The questionnaire was pre-tested and revised prior to baseline data collection. Trained data collectors were not blinded to intervention group status.

A census of all individuals responsible for analysis and response within intervention and control CPH offices was recruited to participate in the study. In total, 31 individuals responsible for analysis and use of VPD data from within the 12 intervention rayons were identified and completed the questionnaire at baseline. All of these individuals, plus an additional 4 who joined or were promoted within the same CPH offices, completed the questionnaire at follow-up post-intervention. Eleven individuals responsible for analysis and use of VPD data from within the three control rayons were identified and completed the questionnaire. Ten of these same individuals completed the questionnaire at follow-up. A total sample size of 87 observations was obtained over both rounds among both intervention groups.

The survey was complemented by a review of CPH records and reports at two points in time after the initiation of the intervention to ascertain whether the intervention package was implemented successfully, and was functioning as intended. The record reviews were conducted within both intervention and control rayons in May 2004 and April 2005 (Figure 2) in order to a) to assess completeness and accuracy of the records in the workbooks distributed to rayon epidemiologists in the program intervention areas; and b) to determine the current level of data analysis and use in other rayons beyond the program pilot area.

Record reviews were using special instruments developed by research team members. Respondents were those professionals who are directly responsible for maintaining all records and conducting the analyses of surveillance data. In addition, all worksheets that had been completed were photocopied for further detailed analysis and review. The worksheet's data aggregation section was considered as accurate if, through a random check, data in the workbook corresponded to those in the primary data sources and there were no mathematical mistakes. The worksheet's logical conclusions section was considered as accurate if analytical conclusions (e.g., which units performed poorly, causes of low coverage, reasons why cases occurred, who belongs to risk groups, barriers to performance) were made based on a complete set of data and logically reflected these data.

In addition, focus group discussions within intervention rayons were conducted post-intervention at two points in May 2004 and April 2005. The following groups of staff members from intervention CPH offices and health care facilities were included in the focus group discussions:

 CPH office epidemiologists

 CPH office directors

 Polyclinic clinicians

 Polyclinic directors

For each group, two focus group discussions were held at each of the two time periods, and the size of the group ranged from five to seven individuals. Guides were developed separately for CPH staff and providers. Participants were mostly the same for the two rounds. The length of the discussion sessions averaged between 2 and 2.5 hours for CPH staff and between 1 and 1.5 hours for Polyclinic staff.

Two researchers conducted each focus group discussion: a moderator who led the discussion and a facilitator who handled all logistics and took notes. The facilitator recorded the personal characteristics of the members making up the discussion, the time, duration, and location. As far as possible, the discussions took place in a setting where the session was not interrupted and people felt that they could voice their opinions freely. Each of the focus group discussions were audio taped and transcribed. The research team analyzed the data using domain analysis, which seeks to create a systematic understanding of a practice by describing and analyzing individual's perceptions, attitudes, and experiences. The research team created a coding scheme using broad categories to organize the data, such as the availability of data, the analysis of data, and the use of analyzed data to carry out public health actions. Using these predefined codes, information was organized and displayed. Notes and selected quotations were translated into English.

### Statistical methods

All statistical analyses were conducted using SAS 9.1. The *intervention group *× *survey round *interaction term in the following model was used to assess the effect of the intervention package on selected outcomes:

Y = β_0 _+ β_1_(intervention group) + β_2_(subject) + β_3_(survey round) + β_4_(intervention group*survey round) + β_6_(sex) + β_7_(age) + β_8_(years of experience) + β_9_(rayon) + *e*.

The *intervention group *× *survey round *interaction term can be interpreted as the relative change in outcome indicators between comparison groups from the baseline to follow-up round. Age, sex and years of experience are included to control for individual-level confounders. Rayon is included to control for a number of community-level differences that may exist between rayons, hypothesized to include such potential confounders as governmental funding, access to health care and differences in VPD rates. The analysis of individual Likert scale outcomes was modeled using the generalized linear model (GLM) for multinomial outcomes. Composite index outcomes were analyzed using linear regression in GLM with the generalized estimating equation (GEE) used to account for correlations between subjects with repeated measures. Differences between intervention groups for bivariate outcomes were assessed using Chi-square. For all outcomes, a two-sided P-value < 0.05 was considered statistically significant.

## Results

### Baseline characteristics

Baseline demographic and employment characteristics were similar among respondents in the intervention and control group at baseline. CPH staff respondents responsible for analysis and use of VPD data included directors, deputy directors, epidemiologists and parasitologists. The majority of both intervention (90.3%) and control (81.8%) respondents were female (*X*^2 ^= 0.560, *P-value *= 0.4543). The mean age of respondents was 43.1 and 49.8 years in the intervention and control groups, respectively (t-test for unequal variance = 1.77, *P-value *= 0.0985), while their mean years of professional experience followed at 13.3 and 19.0, respectively (t-test for unequal variance = 1.27, *P-value *= 0.2267). While similar, respondents in the control group responded with greater agreement across all five outcome indicators measuring analysis and use of VPD data compared to those in the intervention group at baseline (Table 2), three of which were significantly different [Perceived ability to perform data analysis (t-test for unequal variance = 5.40, *P-value *< 0.0001); motivation to perform data analysis (t-test for unequal variance = 3.12, *P-value *= 0.0057); and perceived value of using analyzed VPD data for decision-making (t-test for unequal variance = 3.66, *P-value *= 0.0021)].

### Quantitative evaluation results

**Figure 3:**
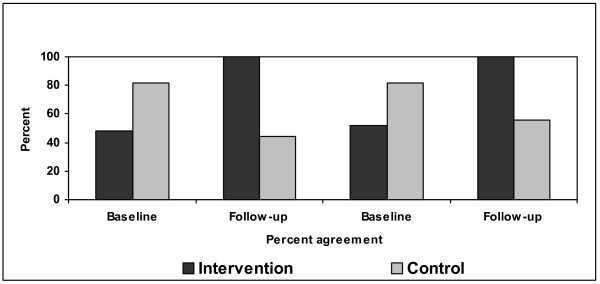
**Percent agreement that there exist written guidelines for analysis and use of vaccine-preventable disease (VPD) data at the Centers for Public Health, by intervention group and survey round**. *Proportion respondents agree there are written guidelines to help guide analysis of VPD data increase significantly within the intervention group (n = 66; *X*^2 ^= 23.86; *P-value *< 0.0001). ^†^Proportion respondents agree there are written guidelines to help in decision-making based on analyzed VPD data increase significantly within the intervention group (n = 66; *X*^2 ^= 21.92; *P-value *< 0.0001).

The survey and record review results suggest that the intervention package was implemented as planned within the 12 intervention rayons (Figure 3). The survey results show that while there was a decrease among respondents within control rayons who stated there were written guidelines for analysis and use of VPD data for decision-making, the proportion of respondents in the intervention areas who stated these guidelines existed significantly increased from 48.4% and 51%, respectively, at baseline to 100% at follow-up (Guidelines for analysis: *X*^2 ^= 23.86, *P-value *< 0.0001; guidelines for using data for decision-making: *X*^2 ^= 21.9, *P-value *< 0.0001). Project records also show that the financial standards system was implemented as planned as evident by the fact that the regional CPH office in Imereti reported receiving and processing reimbursement requests from each of the twelve districts during the intervention period.

Following the largely ambivalent responses to these three questions on the availability of quality VPD data, the mean score for this index increased only modestly by 0.53 between the baseline and follow-up among the intervention group, as compared to essentially no change among the control group (Table 2). Accordingly, the effect of the intervention package was not shown to be statistically significant (*P-value *= 0.1664) on the perception of the availability of quality surveillance data.

However, the effect of the intervention package was found to have significantly improved self-perceived capacity to perform data analysis (*P-value *= 0.0003), as the mean score for this index increased by 0.50 among the intervention group while decreasing by 0.70 among the control group pre and post-test (Table 2). The intervention package also had a marginally significant impact (*P *= 0.0579) on the reported motivation to analyze VPD data, as the mean Likert scale responses in the intervention group increased by 0.41 while the mean responses decreased by 0.59 in the control group pre and post-test.

Results from the record review also found improvements in the analysis of data, but suggest that analytical accuracy varied widely across the intervention rayons. For example, the proportion of rayons that produced accurately completed worksheets, as verified through comparison with original source data, ranged from 8.3% on some worksheets to 83.3% on others. While these statistics stand in contrast to survey results reported above on the perceived capacity and motivation to perform analysis, the mean completion rate of workbook sheets increased from 49.2% (Standard deviation (SD) = 13.3) in 2003 to 77.5% (SD = 11.14) in 2004, while the mean completion rate of workbook sheets considered accurate increased from 29.1% (SD = 12.58) in 2003 to 50.0% (SD = 21.52) in 2004 (2003 data not shown, calculated in 2003 since the introduction of the intervention from October to December 2003). No documented evidence of analysis of VPD data was found at the three control rayons during the record reviews. While the actual use of the workbook varied widely across rayons, most rayons (83.3%) did produce evidence that the analysis of surveillance data was used to produce statistical reports, while no such evidence was produced in the three control rayons.

While point estimates and corresponding coefficients of the *intervention group *× *survey round *interaction term were in hypothesized directions, the intervention package did not have a significant impact on the perceived value of using analyzed VPD data or the motivation to use such data for decision making.

### Qualitative evaluation results

The results of the focus group discussions of CPH and health care facility staff point to a number of improvements as a result of the intervention. These included: improved knowledge about current regulations at both the local and health facility levels; clarification of the roles and responsibilities of staff at regional, rayon, and health facility levels; an increased sense of job responsibility regarding roles in the surveillance system; and increased ability of CPH staff to carry out critical surveillance functions.

Despite these improvements, the qualitative component of the research highlights several potential barriers that prevent the use of the intervention tools to their fullest extent [see additional file 1]. The barriers mentioned include: an insufficient availability of quality surveillance data from subordinate health facilities; the unavailability of phones and electricity in health facilities and CPH offices; low levels of health care utilization; poor reporting of data from some private providers; a common perception that evidenced-based recommendations for public health actions will likely not be adopted by those at higher levels; the lack of CPH authority to impose penalties on low-performing health facilities; and limitations of government resources to carry out surveillance and response.

## Discussion

The results of the study suggest that the intervention package, which included guidelines, the job aid workbook, training, and a financial reimbursement system, were implemented as planned and led to expected improvements in the availability and analysis of surveillance data but not in the use of data for public health response, as assessed through the use of a pre-post quasi-experimental design. As suggested by the results of the focus group discussions with district and health facility staff, external factors, particularly those that operate at the health systems level, played an important role in limiting the effectiveness of the intervention in strengthening the availability and use of data for programmatic and epidemiological responses at the local level.

That the intervention did not result in substantial improvements in the use of data for public health responses during the course of the intervention most likely stems from the problem of weak accountability relationships within Georgia's health system. For example, the process of decentralization that begin in the mid-1990's resulted in fragmentation between health care facilities and CPH offices and a lack of clarity about which level bears responsibility for some key surveillance and response functions, such as outbreak investigations. In order to improve the effectiveness of surveillance and response and other HIS interventions, it will be critical to link the intervention to reforms in management processes and organization culture [11]. In particular, it will be important to assess the various accountability roles that actors in the surveillance and response system play, and to develop accountability-strengthening strategies to help ensure the maximum effectiveness of the intervention in promoting evidence-based decision-making [12]. This is critical given that the government has proceeded to scale up the intervention to the rest of Georgia.

Health systems barriers are also likely to play a critical role in surveillance and response systems and in other types of HIS interventions, both in Georgia and in other countries [1,2,6-8,13-16]. In order to strengthen HIS, governments, international agencies and donors should consider how health systems factors influence the effectiveness of HIS investments not only on the availability and quality of information, but also on the value of the information to those who are expected to use it.

The results of this study should be treated with caution for several reasons. First, despite the census nature of the district-level data collected, limitations in sample size greatly limited the statistical power of the analysis which allowed the detection of only a 20% or greater change in the outcome between treatment groups across study rounds, assuming a two-sided test with alpha set at 5%, the baseline proportion of self-reported motivation to use and analyze data set at 60% (agree-strongly agree), repeated-measure design and 80% power. Secondly, because the intervention package was implemented within the Imereti region as a full coverage program, rayons were not randomly assigned to intervention and control groups, which limit the internal validity of the study design. Third, despite the self-administered questionnaire format, social desirability bias was still possible. This may explain why no statistical impact was detected for the indicator measuring self-reported motivation to use analyzed surveillance data, which was very high at baseline within both the intervention and control groups, and continued to be high in both follow-up survey rounds. In this instance, it is likely that the results were biased towards the null hypothesis of no change. Fourth, this study design prevented us from assessing to what extent each intervention component contributed to improvements in analysis and response. Thus, lack of changes, or detected changes, in various aspects of analysis and response may have been due to limitations in the measurement tools used.

Our findings regarding the health system constraints to strengthening public health surveillance and response functions are consistent with findings of previous researchers who have investigated the effectiveness of strategies to improve the use of data for decision-making in developing countries. For example, in a study of the effectiveness of a multi-faceted strategy supported by USAID and the US Centers for Disease Control and Prevention to improve capacity to use data for decision making at the local-, district-, regional-, and national-levels in four counties with decentralized health system (Bolivia, Cameroon, Mexico, and the Philippines), Pappaioanou et al found that although substantial improvements in data use were achieved, the sustained use of evidence-based public health in the long term requires "the creation of a data-use culture and a behavior change in those involved with the decision-making environment" [17]. A review of public health surveillance and response strategies also finds that health system factors including many identified in our study present substantial challenges in low- and middle-income countries, and that building sustainable programs requires long-term, sustained efforts [6].

The logical implications of such findings are enormous, as they suggest that the process of building effective surveillance and response systems as well as other types of HIS may best be carried out within the context of a broader and long-term health sector reform process that involves not only clarifying health sector priorities, refining policies, and consensus-building in a decentralized context, but also reforming and restructuring institutions through which surveillance and response activities are implemented.

## Conclusion

To strengthen surveillance and response systems in Georgia, as well as in other countries, donor, governments, and other stakeholders should consider how organizational and health systems factors influence investments to improve the availability of data, analysis, and response. Linking the surveillance and response interventions to broader reforms in management process and organizational culture will be critical to ensure that efforts designed to promote evidence-based decision-making are successful, especially as they are scaled up to the national level.

## Competing interests

The author(s) declare they have no competing interests.

## Authors' contributions

DRH, TPE and MD conceived the research and study design, lead the analysis and drafted the manuscript. MD also supervised data collection and assisted with drafting of the manuscript. EAS performed data analysis and assisted with drafting of the manuscript. NR performed data collection and performed analysis of qualitative data. All authors read and approved the final manuscript.

## Pre-publication history

The pre-publication history for this paper can be accessed here:



## Supplementary Material

Additional file 1 Quotations from focus group discussions regarding health system barriers Click here for file

## Figures and Tables

**Table 1:** Composite Indices for measuring underlying constructs of attitudes towards analysis and use of vaccine-preventable disease (VPD) data

Questions*	**Composite Index (Outcome indicator)**	**Cronbach coefficient alpha (raw)**	**Mean Score**	**Standard deviation**
Subordinate health facilities and labs report their surveillance data in a timely manner.				
Reports submitted by subordinate health facilities are fully completed	Perceptions of availability of quality VPD data	0.75	2.82	0.67
I have confidence that the surveillance data reported by subordinate health facilities are accurate				

I possess sufficient skills to analyze and interpret surveillance data				
I feel fully capable of carrying out analysis of surveillance data	Perceived capability to perform analysis of VPD data	0.88	3.71	0.84

Epidemiological data are essential for providing effective surveillance of vaccine-preventable diseases in my rayon.				
Data from subordinate health facilities must be analyzed in order to be useful.				
I place great importance on providing feedback to subordinate health facilities based on the data that I routinely analyze.	Perceived value of using analyzed VPD data for decision-making	0.79	4.21	0.42
Analysis of surveillance data is useful because it provides a basis for decision-making.				
It is important that decisions regarding prevention and control of infectious diseases be based on solid evidence.				

Results based on baseline data pooled among intervention and control respondents (n = 42), with data imputed for non-response by taking the average value of the questions that were answered.

*Likert scale responses at 5 levels (1–5) from strongly disagree to strongly agree.

**Table 2:** Results of regression analyses assessing the impact of the job aid intervention on the five primary outcome indicators for analysis and use of vaccine-preventable disease data

**Outcome indicator**	**Mean values from Likert scale**			**Regression results**			
n = 87 observations	***Intervention group***	***Pre-test***	***Post-test***	Model*	***Interaction coefficient* ^†^**	***Standard error***	***P-value***
1. Perceptions of availability of quality VPD data	Intervention	2.75	3.28	Linear regression	0.6533	0.4722	0.1664
	Control	3.00	3.18				
2. Perceived capability to perform analysis of VPD data	Intervention	3.40	3.90	Linear regression	1.1191	0.3110	0.0003
	Control	4.50	3.80				
3. Likert scale question measuring motivation to carry out analysis	Intervention	3.16	3.57	Multinomial regression	-2.4354	1.2843	0.0579
	Control	4.09	3.50				
4. Perceived value of using analyzed VPD data for decision-making	Intervention	4.08	4.09	Linear regression	0.2824	0.2205	0.2004
	Control	4.56	4.38				
5. Likert scale question measuring perceived motivation to use surveillance data	Intervention	3.81	3.92	Multinomial regression	-1.4828	1.4084	0.2924
	Control	4.18	3.60				

VPD = Vaccine preventable disease

*Regression models included: intervention group, subject, survey round, intervention group*survey round interaction term, and controlled for sex, age, years of experience and rayon. Linear and multinomial regression performed using generalized linear model in SAS (Proc Genmod), with generalized estimating equation (GEE) used to account for correlations between subjects with repeated measures.

^†^Impact of intervention assessed by intervention group*survey round interaction term.
