# Supplementary material for: Health system barriers to strengthening vaccine-preventable disease surveillance and response in the context of decentralization: evidence from Georgia
Source: BMC Public Health. 2006 Jul 5;6:175. doi: 10.1186/1471-2458-6-175 (PMC1526426; doi:10.1186/1471-2458-6-175)
Supplement: Additional file 1 — Quotations from focus group discussions regarding health system barriers [file 1471-2458-6-175-S1.doc]

**Additional File 1: Quotations from focus group discussions regarding health system barriers**

Availability of quality information

*“The problem of communication between village ambulatories and rayon CPHs still remains unchanged. This problem is especially critical for remote and high mountainous villages. Due to this reason number of cases are still notified with delay” (Polyclinic provider)*

*“There are no penalties for those who do not report the surveillance data. We are limited” (CPH epidemiologist)*

*“Financial motivation is essential. Due to the low salaries providers sometimes do not perform their routine work perfectly” (CPH epidemiologist)*

*“People usually do not seek care at official facilities due to they cannot afford the care, thus significant number of cases are not registered” (CPH epidemiologist)*

Analysis of information

*”Quality of data still remains a problem. In particular, private practitioners still violate regulations which affect the quality of data” (CPH epidemiologist)*

*"Results of the analysis are not followed by appropriate response actions” (CPH epidemiologist)*

Use of information

*“Everything is defined by economic situation. Whatever high quality analysis is done if they are not supported with financial means nothing will happen. Resources are very limited” (CPH epidemiologist)*

*“CPH offices have been separated from the sanitary inspection. As a result, they have lost authority as their responsibilities have been cut down” (CPH Director)*

*“Only in the case of a serious epidemic are they (authorities) are concerned and you have more chances to get support” (CPH epidemiologist)*
